# NFAT5 Isoform C Controls Biomechanical Stress Responses of Vascular Smooth Muscle Cells

**DOI:** 10.3389/fphys.2018.01190

**Published:** 2018-08-23

**Authors:** Maren Zappe, Anja Feldner, Caroline Arnold, Carsten Sticht, Markus Hecker, Thomas Korff

**Affiliations:** ^1^Division of Cardiovascular Physiology, Institute of Physiology and Pathophysiology, Heidelberg University, Heidelberg, Germany; ^2^Medical Research Center, Medical Faculty Mannheim, Heidelberg University, Heidelberg, Germany; ^3^European Center for Angioscience, Medical Faculty Mannheim, Heidelberg University, Heidelberg, Germany

**Keywords:** vascular smooth muscle cells, wall stress, hypertension, transcriptional regulation, NFAT5, TonEBP

## Abstract

Vascular cells are continuously exposed to mechanical stress that may wreak havoc if exceeding physiological levels. Consequently, mechanisms facing such a challenge are indispensable and contribute to the adaptation of the cellular phenotype. To this end, vascular smooth muscle cells (VSMCs) activate mechanoresponsive transcription factors promoting their proliferation and migration to initiate remodeling the arterial wall. In mechanostimulated VSMCs, we identified nuclear factor of activated T-cells 5 (NFAT5) as transcriptional regulator protein and intended to unravel mechanisms controlling its expression and nuclear translocation. In cultured human VSMCs, blocking RNA synthesis diminished both baseline and stretch-induced NFAT5 mRNA expression while inhibition of the proteasome promoted accumulation of the NFAT5 protein. Detailed PCR analyses indicated a decrease in expression of NFAT5 isoform A and an increase in isoform C in mechanoactivated VSMCs. Upon overexpression, only NFAT5c was capable to enter the nucleus in control- and stretch-stimulated VSMCs. As evidenced by analyses of NFAT5c mutants, nuclear translocation required palmitoylation, phosphorylation at Y143 and was inhibited by phosphorylation at S1197. On the functional level, overexpression of NFAT5c forces its accumulation in the nucleus as well as transcriptional activity and stimulated VSMC proliferation and migration. These findings suggest that NFAT5 is continuously expressed and degraded in resting VSMCs while expression and accumulation of isoform C in the nucleus is facilitated during biomechanical stress to promote an activated VSMC phenotype.

## Introduction

Arteries provide a hierarchically organized network for the distribution of blood throughout the organism and are exposed to variable physical forces as generated by blood flow and blood pressure. The form and architecture of the arterial wall corresponds to its function (i.e., control of vascular tone and organ perfusion) while retaining some plasticity that allows for adaptive remodeling in response to altered biomechanical load. In fact, disturbed (non-laminar) blood flow and chronically elevated blood pressure are leading causes of arteriosclerosis and arterial remodeling (Intengan and Schiffrin, 2000; AlGhatrif and Lakatta, 2015) which are thought to be preceded by the mechanoactivation of vascular endothelial and smooth muscle cells. The latter retained the ability to switch their phenotype from quiescent/contractile to activated/synthetic which discriminates them from other muscle cells. Activated vascular smooth muscle cells (VSMCs) are characterized by an increased proteolytic activity and production of extracellular matrix (e.g., collagen type I) to structurally strengthen the arterial wall. This is accompanied by their proliferation which drives arterial thickening (Intengan and Schiffrin, 2001; AlGhatrif and Lakatta, 2015).

Wall stress-mediated mechanoactivation of arterial VSMCs poses as one critical step within this cascade of events and evokes an extensive adaption of the transcriptome. To this end, the activity of transcriptional regulators needs to be altered. For instance, myocardin - a coactivator of the serum response factor (SRF) becomes inactivated upon exposure to hypertension or biomechanical stretch and this attenuates the expression of gene products belonging to the contractile apparatus (Pfisterer et al., 2012). In contrast, activator protein-1 (AP-1) controls expression of a number of stress response genes, including those controlling cell proliferation (Ahn et al., 2002) inflammation (Holschermann et al., 2006; Demicheva et al., 2008) and matrix-proteolysis (Bergman et al., 2003) under these conditions.

Another transcription factor that controls the VSMC phenotype is nuclear factor of activated T-cells 5 (NFAT5 or TonEBP - tonicity enhancer binding protein) - a member of the Rel family of transcription factors (Lopez-Rodriguez et al., 1999b; Halterman et al., 2012). Originally described as a hypertonicity-responsive transcription factor that orchestrates cellular homeostasis (Miyakawa et al., 1999), NFAT5 also regulates the phenotype of growth factor-stimulated VSMCs (Halterman et al., 2011). Furthermore, we revealed that NFAT5 is activated in VSMCs exposed to biomechanical stretch (Hodebeck et al., 2014) to control the expression of gene products such as the cytoskeletal filament kappa-actin and the extracellular matrix protein tenascin C. However, combinations of the first five exons of the NFAT5 gene may result in four splice variants (Dalski et al., 2000) encoding for proteins with three different N-terminal ends (Lopez-Rodriguez et al., 1999a). While regulatory mechanisms of NFAT5 expression and activation have been extensively studied in the context of hypertonicity (Dahl et al., 2001; Eisenhaber et al., 2011), it is so far unknown whether comparable mechanisms regulate NFAT5 in biomechanically stressed VSMCs. To this end, we investigated mRNA expression and protein accumulation of NFAT5 in mechanostimulated VSMCs and studied its functional relevance for cellular activity.

## Materials and Methods

### Cell Culture

Isolation of human arterial smooth muscle cells (HUASMCs) was approved by the local Ethics Committee (Heidelberg, Germany; DFG reference KO2254/8-1-336/2005) and conformed to the principles outlined in the Declaration of Helsinki (1997). Parental consent was obtained for isolation of cells from the umbilical cords of the newborns. HUASMCs were freshly isolated from the explanted media of individual umbilical arteries and cultured in DMEM supplemented with 15% FCS. Purity of the cultured cells was routinely controlled by immunoblot- or immunofluorescence-based techniques detecting smooth muscle actin or smooth muscle myosin heavy chain. For biomechanical stimulation, HUASMCs were grown on collagen I-bonded BioFlex^®^ plates (Flexcell^®^ International). Stretch was typically applied at a frequency of 0.5 Hz and an elongation of 0.5–13% for 24 h by using a Flexcell^®^ FX-5000^TM^ Tension System. In addition, cells were exposed to the following reagents for 24 h: 2-bromopalmitate (2-BP) 100 μM (Sigma-Aldrich), 17-Octadecanoic acid (17-OCA) 25 μM (Merck). If DMSO was utilized as solvent, control cells were adequately treated with corresponding concentrations as solvent control.

### Transfection of DNA Plasmids and Viral Transduction

Plasmids (pCMV6) encoding for Myc-DDK-tagged *NFAT5* were bought from Origene (NFAT5_isoform A: RC219340; NFAT5-isoform C: RC216142). Prior to transfection, HUASMCs were washed and 1 ml medium (DMEM+15% FCS) was added to the cells. An cell area of 10 cm^2^ was exposed to 4.5 μg plasmid DNA (*NFAT5a/c*) diluted in 50 μl Opti-MEM I -medium together with 10 μl polyethylenimine (PEI, 1 μg/ml) for 15 min at room temperature. The medium was changed 4 h after incubation at 37°C. Cells were utilized for experiments 24 h after transfection. For enhanced transfection rates, overexpression of *NFAT5c* was achieved by transducing VSMCs with an adenoviral vector expressing *NFAT5c* under a CMV promoter (Sirion Biotech, Germany).

### Mutagenesis-PCR

For mutation of the *NFAT5c*-plasmid, the QuikChange II XL Site-Directed Mutagenesis Kit (Agilent Technologies) was used according to the manufactor’s instructions. Mutation primers were designed as follows: mut1197E for 5′-ggg gtt gtg cct gtt ctt gct caa gca tag gag tct gga tg-3′, rev 5′-cat cca gac tcc tat gct tga gca aga aca ggc aca acc cc-3′; mut143A for 5′-cag gtg gtg gtg aga tgg cca aga ctg tgt gcc tct-3′, rev 5′-aga ggc aca cag tct tgg cca tct cac cac cac ctg-3′.

### Transfection With siRNA

Human arterial smooth muscle cells were transfected with short interfering RNA directed against *NFAT5* (5′-*CCA GTT CCT ACA ATG ATA A*-3′). As control commercially available siGENOME Non-Targeting siRNA (Thermo Fisher Scientific, Bonn, Germany) was applied. For each well of a 6-well plate, 3 μg of siRNA was diluted in Opti-MEM I (Invitrogen) together with 3 μl of MATra-si reagent (IBA, Göttingen, Germany) to give a final volume of 200 μl. After mixing and incubating for 20 min at ambient temperature, the solution was added onto the cells which had been cultivated in 2 ml Opti-MEM I prior to the transfection. Cells were then incubated on a magnet plate (IBA) at 37°C and 5% CO_2_. After 15 min, cells were washed and cultured in normal cell medium for 48 h.

### Genome Array-Based Analyses

Human arterial smooth muscle cells were treated with siRNA against *NFAT5* or non-targeting siRNA and exposed to biomechanical stretch for 24 h (13%, 0.5 Hz). RNA was isolated and processed for human genome array analysis according to manufacturers’ instructions: Gene expression profiling was performed by using the HuGene-1_0-st-v1 array from Affymetrix. Biotinylated antisense cRNA was then prepared according to the Affymetrix standard labeling protocol. Afterwards, hybridization on the chip was performed in a GeneChip Hybridization oven 640 which was then dyed in a GeneChip Fluidics Station 450 and thereafter scanned with a GeneChip Scanner 3000. A custom CDF version 21 with ENTREZ-based gene definitions was used to annotate the arrays. Raw fluorescence intensity values were normalized applying quantile normalization. Differential gene expression analysis was performed with one-way analysis of variance (ANOVA) using the software package JMP10 Genomics version 6 from SAS (SAS Institute). A false positive rate of *a* = 0.05 with FDR correction was taken as the level of significance.

### Analysis of Gene Expression

Total RNA was isolated from the cultured VSMCs using the RNeasy Mini Kit (Qiagen) according to the manufacturer’s instructions. Subsequently, cDNA was synthesized using the Sensiscript Reverse Transcription Kit (205213, Qiagen). Differential PCR analyses to analyze different NFAT5 splice variants were performed based on a protocol reported by Maouyo et al. (2002). Standard PCR was performed using the following primers and conditions: Exon 1: 5′-GCCCTCGGACTTCATCTCATTG-3′, Exon 5: 5′-GGATGCTGCTGAACTGTGTTAC-3′, 5 min at 95°C, 1 min at 95°C, 30 s at 50°C and 30 s at 72°C (35 cycles), and 10 min at 72°C. The PCR products were separated on a 1.5% agarose gel. For quantification, band intensities were measured using the Image J Software Version v1.47v (NIH, Bethesda, MD, United States) and ratios to the internal standard 60S ribosomal protein L32 (RPL32) were calculated. Quantitative real-time RT-PCR for the target sequences was performed in the Rotor-Gene Q (Qiagen) using the LightCycler^®^ 480 SYBR Green I Master Mix (Roche, Mannheim, Germany). Fluorescence was monitored (excitation at 470 nm and emission at 530 nm) at the end of the annealing phase. Threshold cycle (Ct) was set within the exponential phase of the PCR. Quantification of the PCR product was done by using the ΔΔCt method. Amplification of the RPL32 cDNA served as an internal standard. The following primers were used: human *NFAT5* for 5′-AAG AGT GAA GAT GTT ACT CCA ATG GAA G-3′, rev 5′-AAA GTC TGT GCT TGT TCT TGT AGT GG-3′; human *RPL32* for 5′-AGG CAT TGA CAA CAG GGT TC-3′, rev 5′-GTT GCA CAT CAG CAG CAC TT-3′ ; human *TENASCIN C* for 5′-TCA TTG TGG GTC CAG ATA CC-3′, rev 5′-GGA GTC CAA TTG TGG TGA AG-3′.

### Immunofluorescence Analyses of Cultured Cells

Cells were fixed in ice-cold methanol for 15 min and allowed to dry for 20 min. Rehydrated cells were blocked with 0.25% casein and 0.1% BSA in TRIS-buffered salt solution (TBS) for 30 min. Cells were incubated with rabbit anti-NFAT5 antibody 1:100 (sc-13035, Santa Cruz Biotechnology), ms-anti-DDK 1:1000 (TA50011-100, Origene), or rabbit anti-Ki67 1:100 (ab16667, Abcam) or at 4°C overnight. After washing, cells were incubated with donkey anti-rabbit-Cy3 1:100 (Dianova) for 1 h and mounted with Mowiol (Calbiochem). Nuclei were visualized by counterstaining the cells with DAPI (Invitrogen). Fluorescence intensity was recorded using a fluorescence microscope IX83 (Olympus) and quantitated utilizing the TissueQuest Analysis software version 4.0 (TissueGnostics). In brief, nuclei were visualized by DAPI staining, automatically detected and defined as region of interest (ROI). ROI-associated NFAT5 or DDK fluorescence was automatically detected and displayed as percentage fluorescence-positive nuclei with fluorescence signal above background. In experiments requiring transfection of cells, only successfully transfected cells (as evidenced by detection of DDK in the cytoplasm) were included for further analyses.

### Spheroid-Based Analysis of HUASMC Migration

Human arterial smooth muscle cells were suspended in growth medium containing 20% methocel (Sigma-Aldrich). HUSAMCs were cultured as hanging drops for 24 h to form spheroids (500 cells/spheroid). Spheroids were suspended in collagen gels as described earlier (Pfisterer et al., 2014). After 24 h, gels were fixed with 4% formaldehyde and the number and length of the “sprouts” originating from a spheroid were measured (10 spheroids per experimental group) using CellˆD version 3.4 (Olympus).

### Nuclear Protein Extraction

Nuclear protein extraction was performed according to the following protocol: HUASMCs were lysed using buffer I containing 10 mM HEPES, 10 mM KCl, 1 μM EDTA, 1 μM EGTA, 15% Nonidet, protease, and phosphatase inhibitors. After centrifugation (12,000 × *g* at 4°C for 15 min) the supernatant (cytosolic fraction) was transferred to a new tube and stored or immediately used for Western blotting. The remaining pellet was dissolved in 40 μl buffer II consisting of 20 mM HEPES, 400 mM NaCl, 0.01 M EDTA, 0.01 M EGTA, 15% Nonidet and protease and phosphatase inhibitors. Subsequently, this solution was sonicated two times for 5 s at 50 W at 4°C. After centrifugation (12,000 × *g* at 4°C for 15 min) the supernatant containing the nuclear fraction was transferred to a new tube and stored at -80°C or was immediately used. Whole cell lysates were lysed with RIPA-lysis buffer complemented with Halt Protease and phosphatase inhibitor coktail (Thermo Fisher Scientific).

### Western Blot

Protein samples were separated by SDS–PAGE (10%), blotted onto nitrocellulose membranes and analyzed by chemiluminescence-based immunodetection according to standard procedures. The following primary antibodies were used for incubation at 4°C for 24 h: ms-anti-NFAT5 1:500 (sc-398171, Santa Cruz), rb-anti-histone H3 1:1000 (ab1791, Abcam), rb-anti-alpha-Tubulin 1:1000 (2144, Cell Signaling), ms-anti-DDK 1:1000 (TA50011-100, Origene), ms-anti-SMMHC 1:1000 (14-6400-80, eBioscience), rb-anti-calponin 1:2000 (ab46794, Abcam), or rb-anti-myocardin 1:1000 (sc-33766, Santa Cruz), ms-anti-beta-actin 1:5000 (ab6276, Abcam).

### Statistical Analysis

All results are displayed as individual data points of n experiments and the corresponding mean (indicated by blue line) ± SD. Differences between normally distributed values of two experimental groups were analyzed by unpaired Student’s *t*-test with *p* < 0.05 considered statistically significant. Differences between normally distributed values of three or more experimental groups were analyzed by one-way ANOVA followed by a Tukey multiple comparisons post-test with *p* < 0.05 considered statistically significant.

## Results

### Mechanoactivation of Human VSMCs Promotes NFAT5 mRNA Synthesis

**FIGURE 1:**
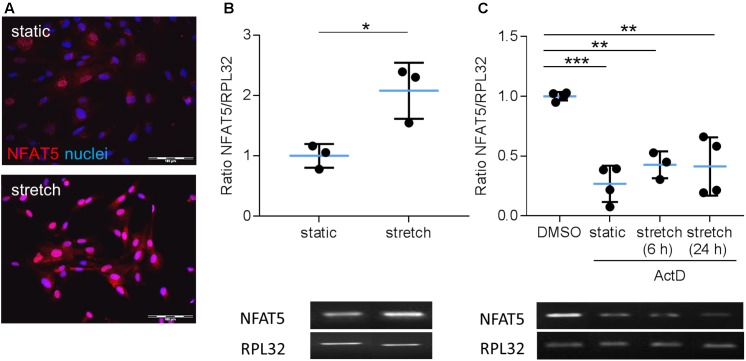
Analysis of NFAT5 mRNA expression in VSMCs. Localization of NFAT5 protein was detected by immunofluorescence (**A**, scale bar: 100 μm) and NFAT5 mRNA expression was analyzed by semi-quantitative PCR (**B**, ^∗^*p* < 0.05, *n* = 3, unpaired, 2-tailed Student’s *t*-test; the expression of the housekeeping gene RPL32 served as internal standard) in resting and stretch-stimulated (24 h) cultured HUASMCs. After treatment (24 h) with actinomycin-D NFAT5 mRNA expression declined (**C**, ActD; ^∗∗∗^*p* < 0.001, ^∗∗^*p* < 0.01, *n* = 4; one-way ANOVA and Tukey post-test).

**FIGURE 2:**
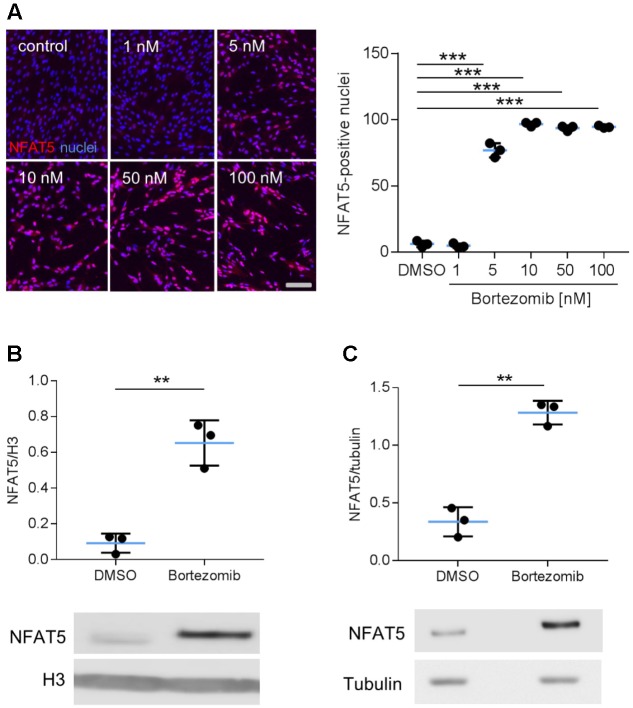
Analysis of NFAT5 protein abundance upon inhibition of the proteasome. HUASMCs were treated (24 h) with increasing concentrations of bortezomib and NFAT5 was detected by immunostaining. The percentage of NFAT5-positive nuclei were quantified by automated image analysis (**A**, ^∗∗∗^*p* < 0.001, *n* = 3, one-way ANOVA and Tukey post-test; scale bar 100 μm). NFAT5 protein abundance was determined after treatment with 10 nM bortezomib (24 h) in nuclear **(B)** and cytosolic (**C**) fractions of cell lysates (**B,C**, ^∗∗∗^*p* < 0.001/^∗∗^*p* < 0.01 vs. DMSO, *n* = 3, unpaired, 2-tailed Student’s *t*-test; histone H3 and tubulin served as loading controls).

Earlier findings suggested a general influence of NFAT5 on the responses of VSMCs to biomechanical stretch (Hodebeck et al., 2014; Scherer et al., 2014). In cultured human arterial smooth muscle cells this stimulus led to a rapid nuclear accumulation of NFAT5 originating from a low baseline level (**Figure 1A**) as well as an increase in NFAT5 mRNA levels (**Figure 1B**). Here, we intended to study mechanisms involved in the control of NFAT5 activity under these conditions and initially analyzed whether biomechanical stress stimulates *de novo* synthesis of NFAT5 mRNA. To this end, mechanoactivated VSMCs were exposed to actinomycin D that forms stable complexes with double-stranded DNA to inhibit DNA-dependent RNA synthesis. This treatment diminished both baseline and stretch-induced mRNA expression of NFAT5 (**Figure 1C**). Interestingly, despite the robust baseline NFAT5 mRNA expression only low levels of NFAT5 protein were usually detected in cultured VSMCs (**Figure 1A**). Thus, we hypothesized that in resting cells NFAT5 may be subject to degradation by the proteasome. To investigate this possibility, VSMCs were treated with increasing concentrations of bortezomib - an inhibitor of the 26S proteasome in therapeutic use (Richardson et al., 2003). As a consequence, NFAT5 accumulated in the cytoplasm and nuclei of VSMCs as evidenced by automated immunofluorescence and immunoblot analyses (**Figure 2**).

### Biomechanical Stress of VSMCs Stimulates Expression of the NFAT5 Isoform C

**FIGURE 3:**
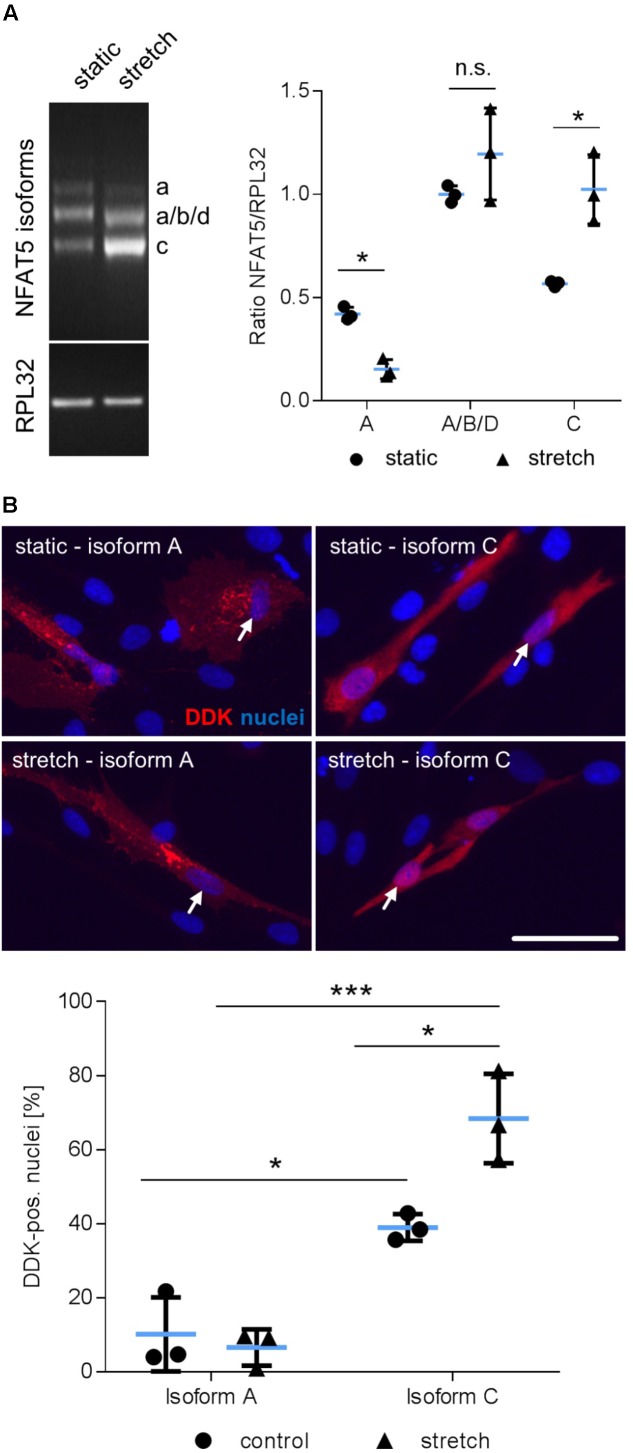
Biomechanical stretch induces NFAT5c translocation into the nucleus. Expression of NFAT5 isoforms after stretch was quantitated by semi-quantitative PCR (**A**, ^∗^*p* < 0.05 vs. control, *n* = 3, unpaired, 2-tailed Student’s *t*-test, n.s.: not significant). DDK-specific immunostaining was used for detection of NFAT5 isoform localization in HUASMCs exposed to biomechanical stretch for 24 h, white arrows indicate localization of the nuclei, scale bar: 50 μm). Automated quantification of DDK-positive nuclei in transfected cells 24 h after stretch (**B**, ^∗∗∗^*p* < 0.001, ^∗^*p* < 0.05, *n* = 3, one-way ANOVA and Tukey post-test).

Considering that NFAT5 is translated from several transcript variants (Dalski et al., 2000, 2001), we further delineated the transcript variant that is expressed in mechanoactivated VSMCs. PCR analyses were applied to simultaneously assess the differential expression of the isoforms *NFAT5a, b, c*, and *d* in response to biomechanical stretch. The corresponding technique is based on a protocol developed by Maouyo et al. (2002) and enables simultaneous identification of isoform-specific PCR products generated under identical conditions by size. Stretch stimulation of VSMCs increased the expression of variant *NFAT5c* but decreased *NFAT5a* (**Figure 3A**). To study the properties of the proteins encoded by either *NFAT5a* or *NFAT5c*, VSMCs were transfected with corresponding plasmids (**Supplementary Figure S1**). The transfection protocol was specifically developed to preserve mechanoresponsiveness of the VSMCs thereby accepting low transfection efficiency. For adequate localization of the DDK-tagged NFAT5a/c proteins, automated immunofluorescence image analyses were applied as described earlier (Hodebeck et al., 2014) including transfected cells only. Upon overexpression, NFAT5c but almost no NFAT5a entered the nucleus of control and stretch-stimulated VSMCs (**Figure 3B**). This result was confirmed by exemplary immunoblot analyses (**Supplementary Figure S2**).

### Posttranslational Modifications Are Required for NFTA5c to Enter the Nucleus of Mechanoactivated VSMCs

**FIGURE 4:**
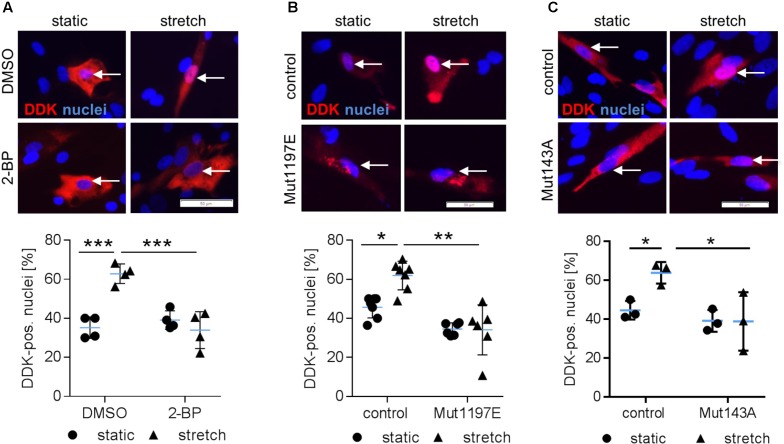
Posttranslational modifications are required for NFAT5c to enter the nucleus of mechanoactivated VSMCs. NFAT5c was overexpressed in HUASMCs and the cells were exposed to biomechanical stretch (24 h). HUASMCs were treated with 2-bromopalmitate (inhibitor of protein palmitoylation, **(A)**, ^∗∗∗^*p* < 0.001, *n* = 4, one-way ANOVA and Tukey post-test, scale bar: 50 μm). Site directed mutagenesis of NFAT5c at serine 1197 (serine → glutamic acid) was performed to mimic phosphorylation at this site (**B**, ^∗^*p* < 0.05, ^∗∗^*p* < 0.01, *n* = 6–7, one-way ANOVA and Tukey post-test, scale bar: 50 μm). Site directed mutagenesis of NFAT5c at tyrosine 143 (tyrosine → alanine) was performed to prevent phosphorylation at this site (**C**, ^∗^*p* < 0.05, *n* = 3, one-way ANOVA and Tukey post-test, scale bars: 50 μm). In all approaches (a-c), localization of NFAT5c in the nuclei was determined by immunofluorescence-based detection of DDK (automated image analysis) in stretch-stimulated HUASMCs.

We next tackled the question which posttranslational modifications of NFAT5c are required to enter the nucleus. In this context, it has been previously shown that palmitoylation as well as phosphorylation of S1197 and Y143 control NFAT5 activity during osmotic stress (Irarrazabal et al., 2004, 2010; Eisenhaber et al., 2011). To investigate if comparable mechanisms control the activity of NFAT5c in mechanoactivated VSMCs, we blocked palmitoylation and mutated the aforementioned phosphorylation sites. Inhibition of NFAT5c palmitoylation by 2-bromopalmitate prevented its nuclear translocation in stretch-exposed VSMCs (**Figure 4A** and **Supplementary Figure S3**). Overexpression of mutated NFAT5c to mimic phosphorylation at S1197 [serine → glutamic acid (E)] limited its entry into the nucleus of stretch-stimulated VSMCs (**Figure 4B**). Phosphorylation of tyrosine 143 appeared to support the stress-induced nuclear entry of NFAT5c given that the phospholytic mutation at this residue [tyrosine → alanine (A)] attenuated NFAT5c nuclear translocation (**Figure 4C**).

**FIGURE 5:**
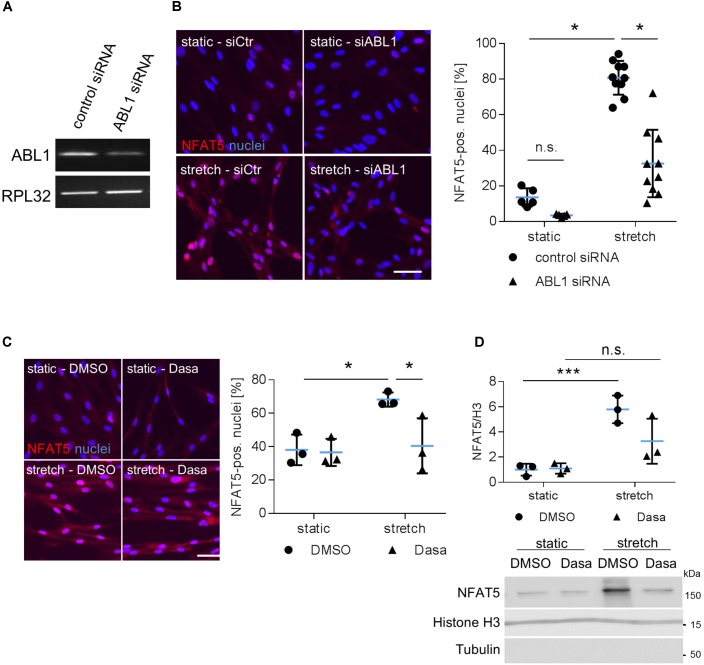
Analysis of nuclear translocation of NFAT5 upon knockdown of ABL1. HUASMCs were transfected with control (scrambled) siRNA or ABL1-specific siRNA (ABL1 Silencer^®^Select Validated siRNA, S864, Ambion, Applied Biosystem, Thermo Fisher Scientific, Darmstadt, Germany) which decreased ABL1 expression by ∼50% as evidenced by PCR analyses **(A)**. Transfected HUASMCs were exposed to biomechanical stretch for 24 h. NFAT5-specific immunofluorescence was detected by automated image analysis to assess the percentage of NFAT5-positive nuclei (**B**, ^∗^*p* < 0.05, one-way ANOVA and Tukey post-test; control siRNA *n* = 5, ABL1 siRNA *n* = 10). Dasatinib (inhibitor of BCR-ABL kinases, 5 nM) treatment of stretch-stimulated HUASMCs decreased the number of NFAT5 positive nuclei as evidenced by automated immunofluorescence analyses (**C**, ^∗^*p* < 0.05, one-way ANOVA and Tukey post-test, *n* = 3). Additionally, HUASMCs were treated with Dasatinib (Dasa; 5 nM) or DMSO for 1 h and exposed to biomechanical stretch for 24 h. Accumulation of NFAT5 in the nucleus was determined in nuclear lysates by immunoblot techniques with (nuclear) histone H3 as loading marker (**D**, ^∗∗∗^*p* < 0.05, n.s.: not significant, one-way ANOVA and Tukey post-test, *n* = 3).

During osmotic stress phosphorylation of NFAT5-Y143 is mediated by c-Abl (ABL1) (Gallazzini et al., 2010). Along these lines, knockdown of ABL1 expression inhibited accumulation of NFAT5 in the nuclei of mechanoactivated VSMCs (**Figures 5A,B**) and prompted us to investigate the impact of the BCR-ABL kinase inhibitor Dasatinib on nuclear translocation of NFAT5. As evidenced by immunofluorescence- (**Figure 5C**) and immunoblot-based (**Figure 5D**) analyses, Dasatinib counteracts an increase in NFAT5 protein in the nuclei of biomechanically stressed VSMCs.

### Overexpression of NFAT5c Activates VSMCs

**FIGURE 6:**
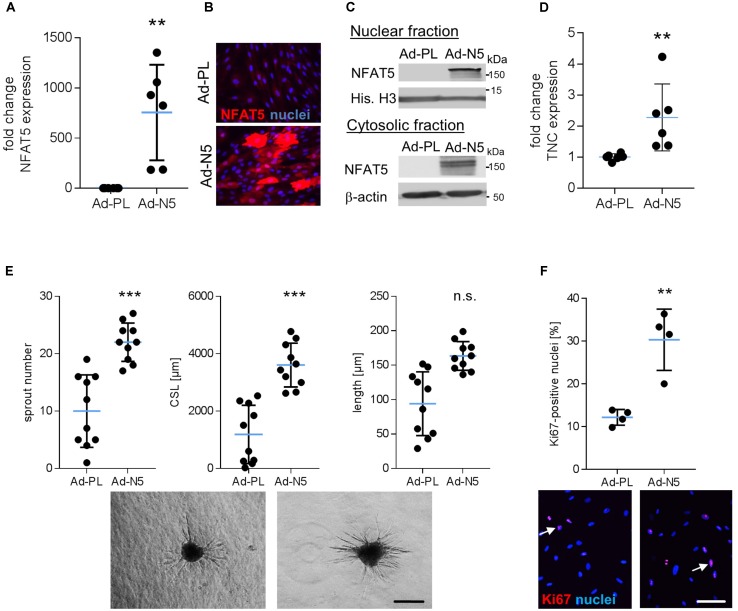
Overexpression of NFAT5c promotes its nuclear accumulation and activates VSMCs. HUASMCs were transduced with adenovirus to overexpress *NFAT5c* (Ad-N5) or (Ad-PL) control plasmid, N*FAT5*-expression was quantitated by qRT-PCR, immunostaining and immunoblot of nuclear and cytosolic lysates 48 h after transfection (**A–C**, ^∗∗^*p* < 0.01 vs. Ad-PL, *n* = 6, unpaired, 2-tailed Student’s *t*-test; histone H3 and β-actin served as loading controls). Expression levels of NFAT5 target gene *TNC* was assessed by qRT-PCR (**D**, ^∗∗^*p* < 0.01 vs. Ad-PL, *n* = 6, unpaired, 2-tailed Student’s *t*-test). The migratory capacity of collagen-embedded *NFAT5c*-overexpressing spheroids was analyzed by assessing the number of sprouts, mean sprout length and cumulative sprout length after 24 h (**E**, ^∗∗∗^*p* < 0.001 vs. Ad-PL, *n* = 10 spheroids/condition, 1 out of 3 independent experiments with comparable results, unpaired, 2-tailed Student’s *t*-test; scale bar: 200 μm). Proliferation of NFAT5c-overexpressing HUASMCs 72 h after transfection by immunostaining of the proliferation marker Ki67 (**F**, ^∗^*p* < 0.05, n.s.: not significant, vs. Ad-PL, *n* = 4, unpaired, 2-tailed Student’s *t*-test, scale bar: 100 μm).

The aforementioned findings suggested that specifically NFAT5c may be involved in the control of VSMC responses to biomechanical stress and may promote an activated/proliferative phenotype under these environmental conditions. To test this hypothesis, we overexpressed *NFAT5c* in VSMCs by applying an adenovirus-based transduction method. As compared to the plasmid transfection technique, utilizing adenoviral vectors substantially increased the percentage of NFAT5 overexpressing cells (**Figures 6A–C**) and allowed for functional analyses but hampered VSMC responses to biomechanical stimuli. In line with the findings shown in **Figure 3B**, NFAT5c accumulated in the nucleus and cytosol (**Figure 6C**) and promoted mRNA expression of its transcriptional target tenascin C (Scherer et al., 2014) under these experimental conditions (**Figure 6D**). On the functional level, NFAT5c amplified both migratory activity (**Figure 6E**) as well as proliferation (**Figure 6F**) of VSMCs.

### Knockdown of NFAT5 Inhibits Adequate Activation of VSMC Upon Exposure to Biomechanical Stress

**FIGURE 7:**
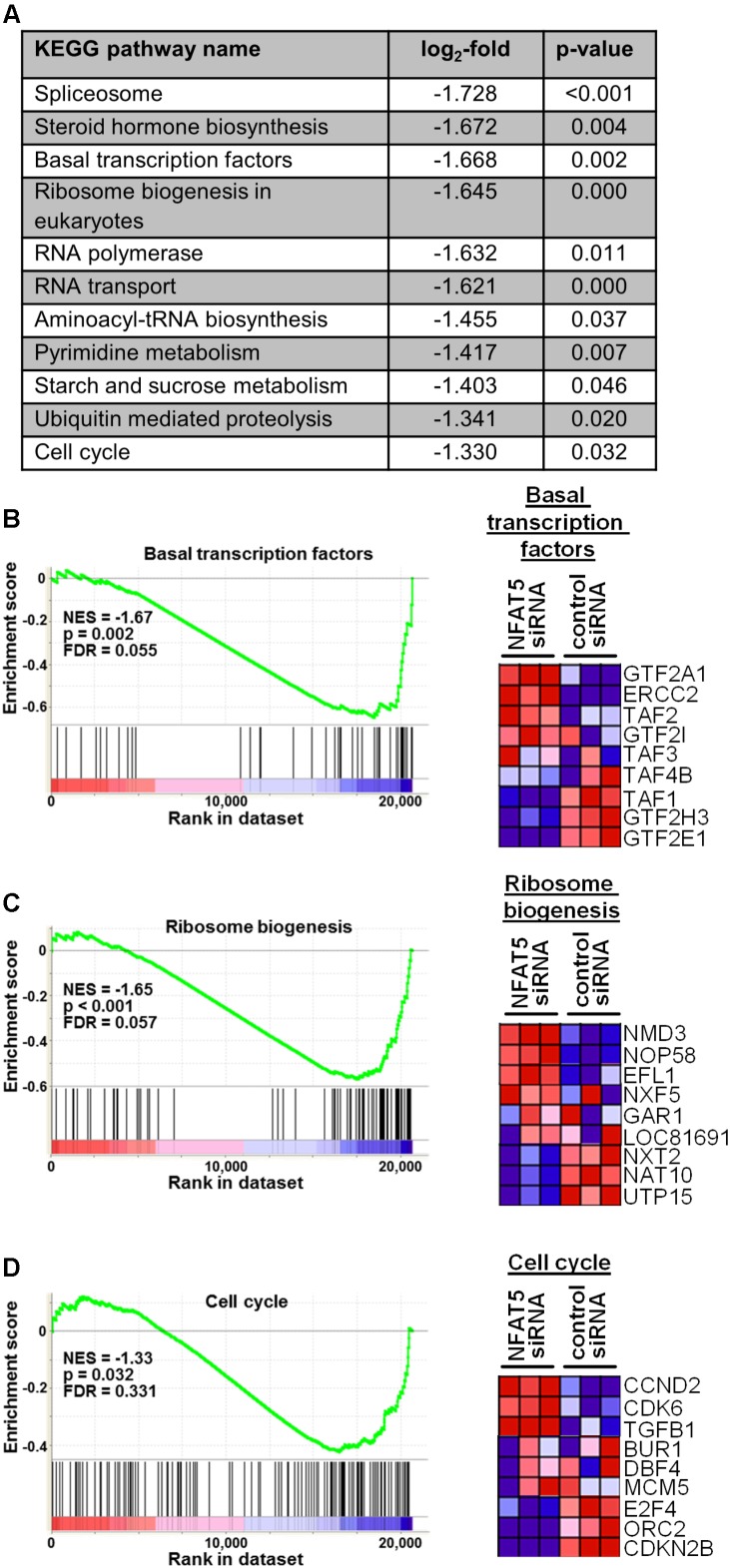
Gene set enrichment analyses (GSEA) based on the KEGG pathway database. HUASMCs from three donors were treated with NFAT5-specific or control siRNA and exposed to biomechanical stretch for 24 h. RNA was isolated and processed for genome array analysis (HuGene-1 0-st-v1 array, Affymetrix). The table **(A)** lists all significantly DOWN-regulated gene sets associated with the indicated pathways/functions (log2-fold regulation, *p* < 0.05 NFAT5 knockdown vs. control, *n* = 3, one-way ANOVA; access# for full microarray data via Gene Expression Omnibus (GEO): GSE106274). Exemplary results of the subsequent gene set enrichment analyses (GSEA) for selected gene sets (enrichment plots) and the corresponding statistical values are shown [**B–D**; NES: normalized enrichment score; p: *p*-value (ANOVA); FDR: false discovery rate]. Additionally, the heat map corresponding to the enrichment plot for cell cycle-associated genes is shown.

**FIGURE 8:**
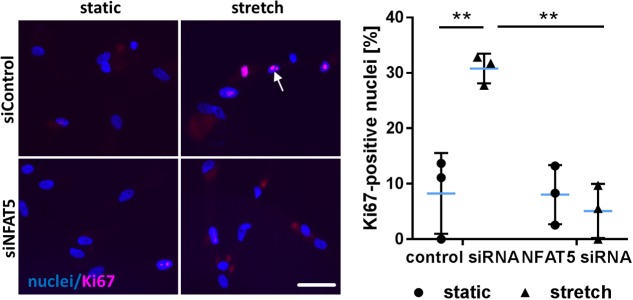
Knockdown of NFAT5 inhibits stretch-induced proliferation of HUASMCs. HUASMCs were treated with NFAT5-specific or control siRNA and exposed to biomechanical stretch for 24 h. Proliferation was assessed by detecting the proliferation marker Ki67 via immunofluorescence techniques (arrow) and determining the percentage of Ki67-positive nuclei (^∗∗^*p* < 0.01, one-way ANOVA and Tukey post-test, *n* = 3; scale bar: 50 μm).

Finally, we tested the general relevance for NFAT5 to orchestrate prototypic responses of mechanoactivated VSMCs. To this end, NFAT5 expression in VSMCs was silenced by siRNA which only minimally affected the expression of genes determining the contractile phenotype [e.g., Myocardin (MYOCD) or SM22 (TAGLN)] under baseline conditions (**Supplementary Figure S4**). Likewise, expression of corresponding genes (MCOCD, TAGLN, ACTA2, CNN1, MYH11, MYL9) was not affected by NFAT5 knockdown in stretch-exposed HUASMCs as evidenced by microarray analyses (data not shown, for details see Gene Expression Omnibus (GEO): GSE106274). However, silencing of NFAT5 promoted downregulation of gene sets controlling fundamental regulatory mechanisms of activated cells such as transcription, translation and cell cycle (**Figure 7**). Consequently, siRNA-mediated knockdown of NFAT5 inhibited the stretch-induced increase in proliferating VSMCs (**Figure 8**) underlining its functional relevance under these conditions.

## Discussion

Chronic biomechanical stress of vascular cells belongs to the most relevant stimuli which promote remodeling of the blood vessel architecture. Within physiological limits, wall stress is well tolerated and to the most part absorbed by components of the extracellular matrix such as elastic fibers which transform the biomechanical load into reversible structural changes. However, wall stress that chronically exceeds these limits may continuously distend or extensively stretch vascular cells and trigger a cascade of responses which may ultimately promote reorganization and proliferation of VSMCs located in the arterial media (Intengan and Schiffrin, 2001). In this context, our former findings suggested that the transcription factor NFAT5 supports a phenotype of VSMCs appropriate to counteract extracellular stressors by adapting the cytoskeleton or promoting motility and proliferation. For instance, NFAT5 controls the expression of actin beta like 2 (*ACTBL2*, kappa-actin) in stretch-exposed VSMCs which affects stress fiber formation and cellular migration (Hodebeck et al., 2014; Scherer et al., 2014). Tenascin C (*TNC*) was identified as another transcriptional target of NFAT5 (Scherer et al., 2014) that is involved in biomechanically evoked vascular remodeling processes (Imanaka-Yoshida and Aoki, 2014) and stimulates proliferation and migration of VSMCs (Ishigaki et al., 2011).

Mechanistically, biomechanical stimuli are sensed through a plethora of mechanisms including ion channels such as TRP channels (Yue et al., 2015), G-protein coupled receptors (Mederos et al., 2008), focal adhesions/cytoskeleton (Kawamura et al., 2003; Kawabe et al., 2004; Lehoux et al., 2005; Liu et al., 2007) or specialized transducer molecules such as zyxin (Ghosh et al., 2015). These initiate further downstream signaling cascades which may promote the activity of protein kinases to alter the expression, stability, localization, or DNA binding affinity of a specific set of transcription factors. For instance, mechanoactivation of VSMCs triggers the activity of the mitogen activated protein (MAP) kinases JNK and ERK1/2 (Hamada et al., 1998; Numaguchi et al., 1999; Kawabe et al., 2004; Kaunas et al., 2006) which phosphorylate transcription factors to either support resting (Pfisterer et al., 2012) or active (Karin, 1995) state of VSMCs.

Nuclear factor of activated T-cells 5 appears to be targeted and activated by similar signaling events in mechanostimulated VSMCs despite being barely detectable on protein level in resting cells which, however, continuously expressed all NFAT5 isoforms on mRNA level. As inhibition of the 26S proteasome promoted accumulation of the NFAT5, we assume that this transcription factor is continuously degraded under homeostasis – a mechanism that would be comparable to other stress-activated transcription factors such as NF-κB or HIF-1a(Thompson et al., 2008) and allow for rapid availability of NFAT5 in cells exposed to biomechanical stress by preventing/overcoming its degradation. In fact, although based on an artificial condition, adenoviral overexpression of NFAT5 appeared to overload the proteasomal machinery and allowed for NFAT5 accumulation and activating modification(s) even in resting VSMCs. Consequently, NFAT5c was sufficient to drive the expression of the transcriptional target tenascin C without any further stimulus under these experimental conditions. Biomechanical stress supported a shift in the mRNA expression of NFAT5 diminishing isoform A and favoring isoform C which appeared to be relevant for VSMCs in this context as only NFAT5c was detectable in the nucleus. As compared to NFAT5a, 76 amino acids extend the N-terminus of NFAT5c (Lee et al., 2003) whose modification may be required to control cellular trafficking of NFAT5. Under osmotic stress, both NFAT5a and NFAT5c have been shown to enter the nucleus of HeLa cells. However, the cell- and stimulus-specific mechanisms controlling the nuclear entry of NFAT5 isoforms are largely unknown. Based on our findings, we assume that at least three separate posttranslational modifications are required to control nuclear translocation of NFAT5c: (i) palmitoylation, (ii) phosphorylation of S1197, and (iii) phosphorylation of Y143. Palmitoylation of NFAT5 (Scherer et al., 2014) is most likely controlled by carnitine palmitoyltransferase I (CPT 1) (Hodebeck et al., 2014) and has been reported to affect the localization of NFAT5a in HeLa cells (Eisenhaber et al., 2011). While palmitoylation usually supports lipid-anchoring of proteins and thus facilitates further posttranslational modifications by membrane-anchored enzymes, we did not observe a membrane-associated localization of NFAT5a/c in VSMCs. Moreover, mutation of individual putative palmitoylation sites of NFAT5c did not influence nuclear translocation of NFAT5c (data not shown) suggesting multiple and compensating modifications of this type.

Phosphorylation of NFAT5 is known as a prerequisite for NFAT5 activity in cells under osmotic stress and may spur nuclear import or nuclear retention by enabling the formation of stable transcriptional complexes (Dahl et al., 2001). Interestingly, phosphorylation of NFAT5c at S1197 prevents its nuclear translocation – a posttranslational regulatory mechanism that also controls NFAT5 transcriptional activity in response to osmotic stress (Irarrazabal et al., 2004). In contrast, phosphorylation of Y143 enables accumulation of NFAT5c in the nucleus which is mediated through c-Abl kinase and phospholipase C gamma 1 as has been reported for cells exposed to osmotic stress (Gallazzini et al., 2010; Irarrazabal et al., 2010). In line with this, the protein kinase inhibitor dasatinib that targets c-Abl kinases interfered with NFAT5 accumulation in nuclei of VSMCs upon exposure to biomechanical stress. As evidenced by earlier findings of our group, this stimulus promotes JNK activity which regulates phosphorylation and nuclear translocation of NFAT5. However, similar to observations made for osmotic stress (Dahl et al., 2001), ERK1/2, p38 MAP kinase or calcineurin were also not affecting biomechanically triggered translocation of NFAT5 (Scherer et al., 2014).

On the functional level, overexpression of NFAT5 isoform C was sufficient to drive the migratory activity as well as proliferation of VSMCs. These functional readouts were utilized as surrogate markers for overall cellular activity and clearly indicate that NFAT5c has the capacity to promote the activated VSMC phenotype which is in line with earlier observations reported for PDGF-BB-stimulated VSMCs (Halterman et al., 2011). In fact, the functional relevance of NFAT5 for biomechanical stress responses of VSMCs was supported by results by an opposite isoform-unspecific approach: Knockdown of NFAT5 in mechanostimulated VSMCs attenuated the expression of transcripts encoding proteins which are involved in the control of gene transcription, translation, and cell cycle. Moreover, proliferation of stretch-stimulated VSMCs was inhibited upon siRNA-mediated silencing of NFAT5.

In a nutshell, NFAT5 emerges as a mechanoresponsive transcription factor whose abundance and activity is regulated on multiple levels to control basic responses of VSMCs to biomechanical stress. In the short run, NFAT5 may thus spur the biosynthesis and motility of VSMCs to rapidly cope with a detrimental environment. In the long run, NFAT5-controlled gene expression may promote VSMC proliferation and matrix synthesis to reorganize their microenvironment for improved structural integrity.

## Author Contributions

MZ, AF, CA, and CS performed the experiments and analyzed the data. MH contributed expertise and wrote the manuscript. TK conceived the study and wrote the manuscript.

## Conflict of Interest Statement

The authors declare that the research was conducted in the absence of any commercial or financial relationships that could be construed as a potential conflict of interest.
