# Supplementary figures and images for: NFAT5 Isoform C Controls Biomechanical Stress Responses of Vascular Smooth Muscle Cells
Source: Front Physiol. 2018 Aug 23;9:1190. doi: 10.3389/fphys.2018.01190 (PMC6115610; doi:10.3389/fphys.2018.01190)

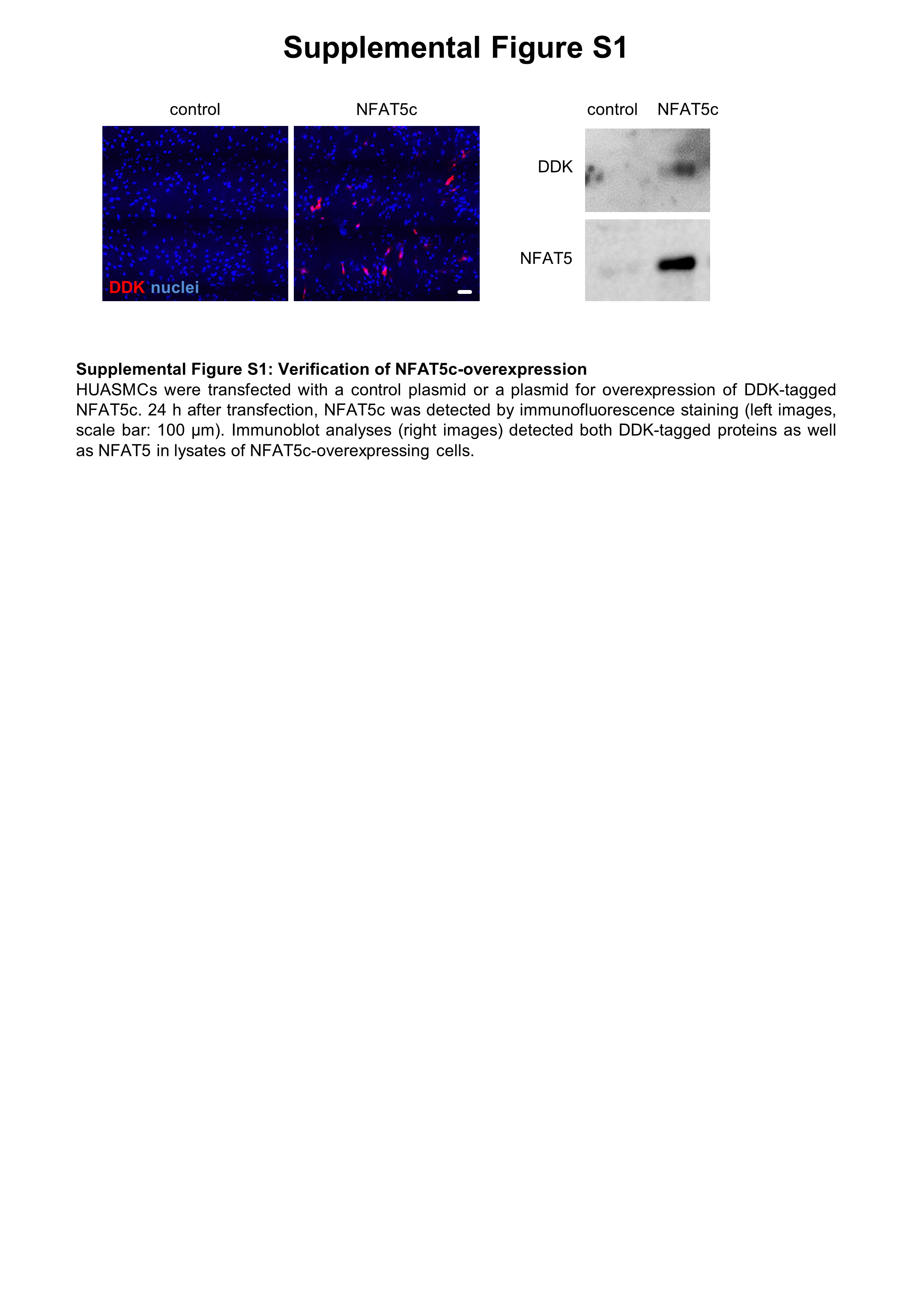

Supplement: Supplementary file 1 [file Image_1.tif]

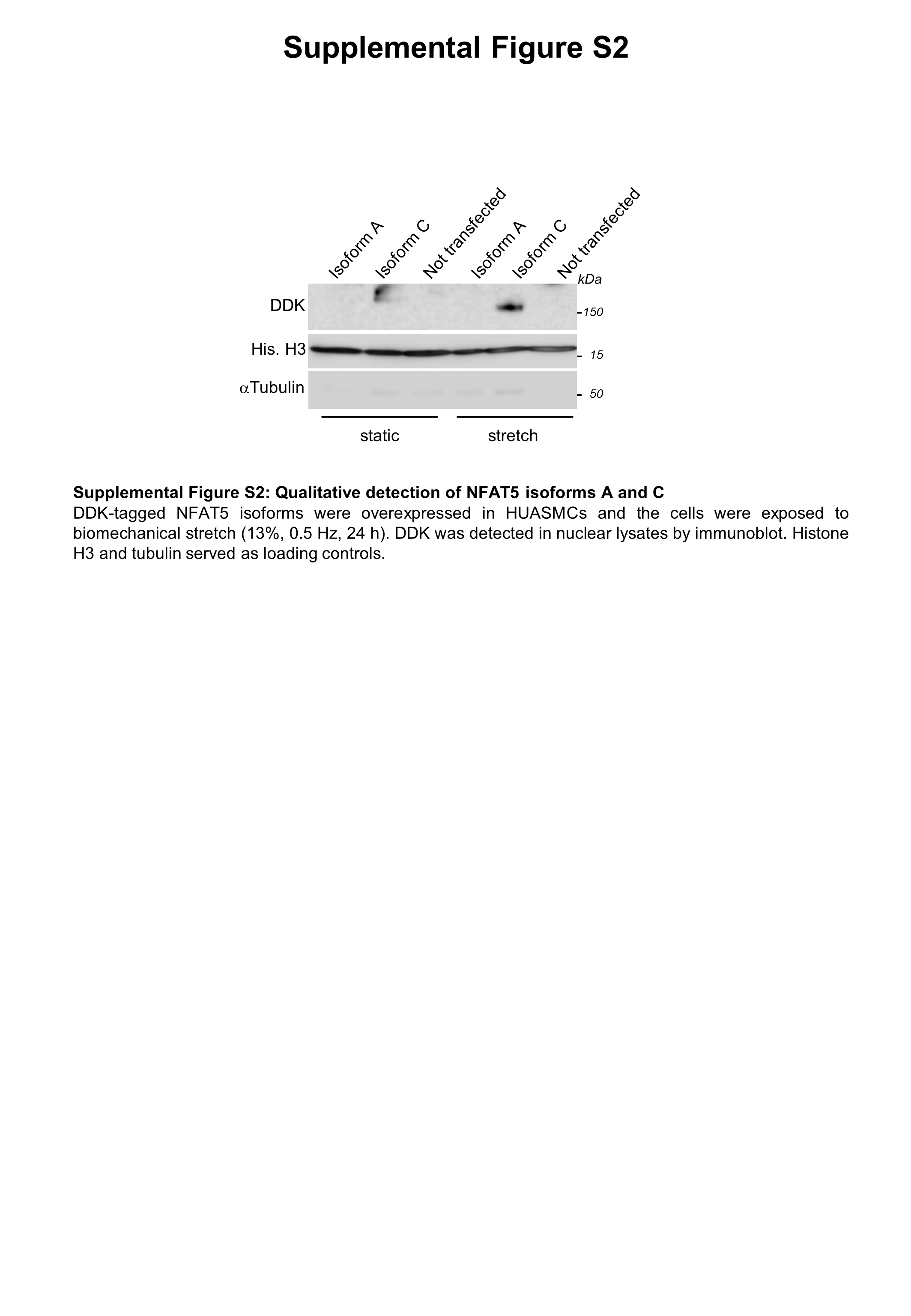

Supplement: Supplementary file 2 [file Image_2.tif]

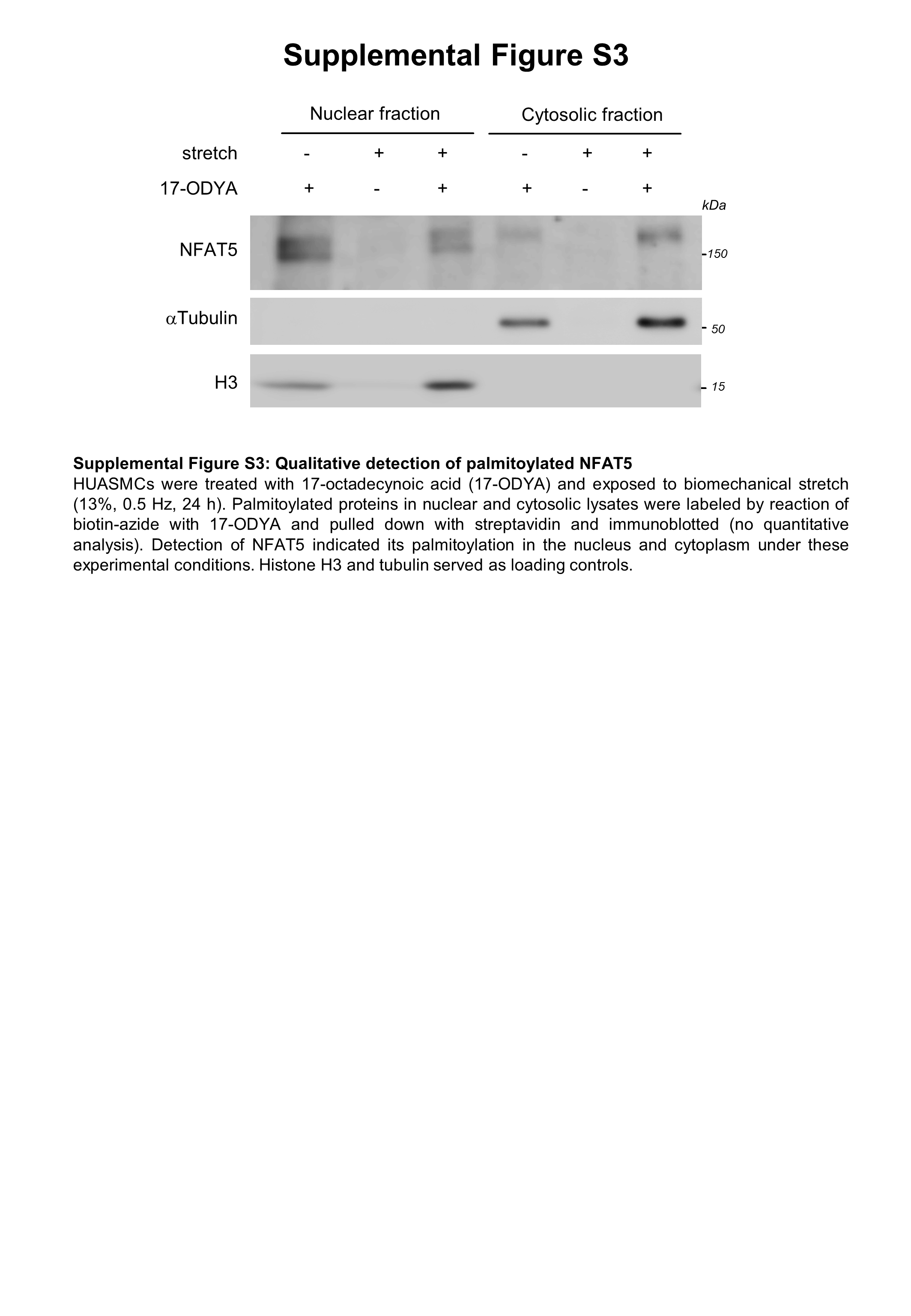

Supplement: Supplementary file 3 [file Image_3.tif]

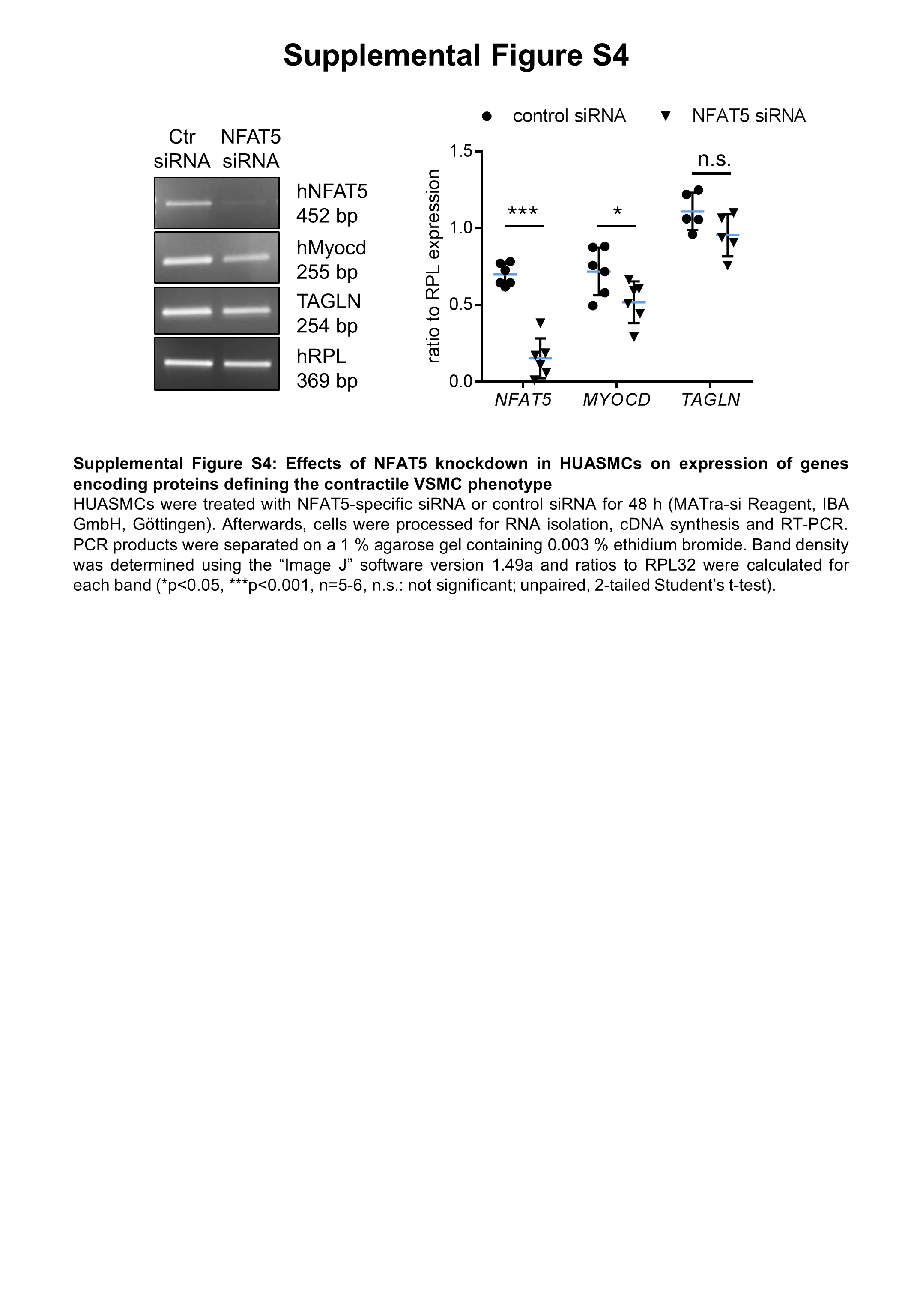

Supplement: Supplementary file 4 [file Image_4.tif]
